# Development of real-time PCR assay for detection of porcine circovirus-like virus P1 in domestic pigs in China

**DOI:** 10.1186/s12917-015-0509-3

**Published:** 2015-09-24

**Authors:** Kong-wang He, Li-bin Wen, Yong-shan Wang, Cheng-ping Lu

**Affiliations:** College of Veterinary Medicine, Nanjing Agricultural University, Nanjing, 210095 China; Institute of Veterinary Medicine, Jiangsu Academy of Agricultural Sciences, Key Laboratory of Veterinary Biological Engineering and Technology, Ministry of Agriculture, National Center for Engineering Research of Veterinary Bio-products, Nanjing, 210014 China

**Keywords:** Porcine circovirus-like virus P1, PCV2, Fluorescence quantitative PCR, Melting curve

## Abstract

**Background:**

The porcine circovirus-like agent P1 is a newly discovered DNA virus with a single-stranded circular genome that is highly homologous to that of porcine circovirus type 2. P1 infection can cause symptoms resembling postweaning multisystemic wasting syndrome. This study aims to develop a rapid, sensitive and specific method to detect P1.

**Results:**

A pair of primers was designed and used to amplify a 119 bp DNA fragment to generate a recombinant plasmid which was served as the standard. A SYBR I qPCR protocol was established using the P1 recombinant plasmid standard and the sensitivity, specificity and stability of this method was analyzed. The results demonstrate a strong correlation with P1 recombinant plasmid titers when virus DNA copy numbers fall in between 10^0^ ~ 10^9^ copies/μL. This method doesn’t detect pseudo rabies, porcine parvovirus or porcine reproductive and respiratory syndrome virus; moreover it can distinguish porcine circovirus type 2 from P1 by melting temperature analysis. Coefficient of variation for each batch of reaction is less than 5 %. The serum virus titers of P1 positive in this study were measured by this protocol to be 10^3^ to 10^7^ copies/mL.

**Conclusions:**

The established qPCR is sensitive, specific, and reliable, which could be a useful tool when applied to quantification of P1 in a variety of samples from infected pigs.

## Background

Porcine circovirus-like agent P1 (P1) is a newly discovered virus. P1 has a circular single-stranded genome of 648 nucleotides, and it is the smallest DNA virus known to date. The whole genome is highly homologous to the genome of porcine circovirus type 2 (PCV2) with the exception of 16 nucleotides. Epidemiological investigation confirmed that the P1 virus has been epidemic in China’s pig farms [1, 2]. To further characterize the P1 virus, we constructed two clones, one with a single copy of P1 virus genome and one with a two copy tandem repeat. It was confirmed that the tandem repeat clone can produce viral particles in transfected PK15 cells, demonstrating that this clone has *in vitro* viral activity. Virus particles are spherical in shape, non-enveloped with a diameter of approximately 25 nm. *In vivo* studies have shown that the tandem repeat P1 clone can infect pigs and the infected animals show symptoms resembling postweaning multisystemic wasting syndrome (PMWS). Clinical manifestations of P1 infection include pale skin, diarrhea and viremia. Generally, lesions include brain hyperemia, bladder mucosal bleeding, inguinal lymph node bleeding, and lung atrophy and bleeding. Histological examines identified diseases including subarachnoid vascular congestion, interstitial pneumonia, myocardial atrophy, tonsil tissue follicular cell hyperplasia, and necrosis of pancreatic cells. P1 nucleic acids and antigens were detected in tissue samples of lung, brain, heart, liver, bladder, pancreas and gonads [3].

The main symptoms of PMWS include wasting and stunting, and sometimes pale skin, jaundice, diarrhea and breathing difficulties [4]. PMWS has become one of the major epidemic diseases that endanger the world’s pig industry [5]. PCV2 is considered a primary pathogen of PMWS [6]. PCV2 belongs to the virus family Circoviridae, and is a small, non- enveloped, single-stranded DNA virus with a genomic size of 1767 or 1768 nucleotides [7–9]. There are three confirmed open reading frames encoded in the PCV2 genome. ORF1 encodes the viral replication enzyme [10], ORF2 encodes the viral capsid protein [11], and ORF3 encodes a protein which is not essential for viral replication but has apoptotic activity [12, 13]. An early study suggested that the virulence of different PCV2 strains correlates with the amino acid sequences of its capsid protein [14]. Several studies followed that focused on ORF2 and based on these studies PCV2 can be divided into different genotypes [15, 16]. Recent studies suggest that PCV2 genotype 1 is more virulent than genotype 2 [17], however, there are discrepancies in the literature [18, 19]. The discrepancy is around the pathogenic characteristics of PCV2, as it is difficult to reproduce the typical symptoms of PMWS with PCV2 alone [20–22]. It is speculated that there may be another contributing factor in the occurrence of PMWS.

The discovery of P1 provides a new etiological explanation for PMWS since the nucleotide sequence of the P1 genome is highly homologous with ORF2 of PCV2 and the encoded capsid protein is also homologous to that of PCV2. The current conventional diagnostic techniques based on the ORF2 of PCV2 may not be able to distinguish PCV2 from P1. Therefore, it is important to establish a rapid, highly sensitive diagnostic method to detect PCV2 and P1 which will aid the prevention and treatment of porcine circovirus-like virus infections. Real-time quantitative PCR has become the preferred method of quantitative detection of nucleic acids due to its’ high sensitivity, specificity and reproducibility as well as being rapid. Among them, the SYBR Green I dye method does not require probe labeling and is highly cost effective and therefore has been more widely used. This study aims to establish an approach based on SYBR Green I dye real-time PCR to accurately and quantitatively detect P1 as well as distinguishing between P1 and PCV2 viruses in animals.

## Methods

### Ethics statement

The use of animal specimens in this study was approved by the Jiangsu Province Animal Regulations (Government Decree No 45). The protocol was approved by the Committee on the Ethics of Animal Experiments of the Institute of Veterinary Medicine, Jiangsu Academy of Agricultural Sciences (JAAS No 20100604).

#### Virus Isolation

Pseudo-rabies virus (PRV), porcine parvovirus (PPV), porcine reproductive and respiratory syndrome virus (PRRSV), porcine circovirus type 2 (PCV2) and serum samples of P1 infected pigs were all isolated and stored in Institute of Veterinary Medicine, Jiangsu Academy of Agricultural Sciences as previously described [23, 24].

### Primers

**Fig. 1:**
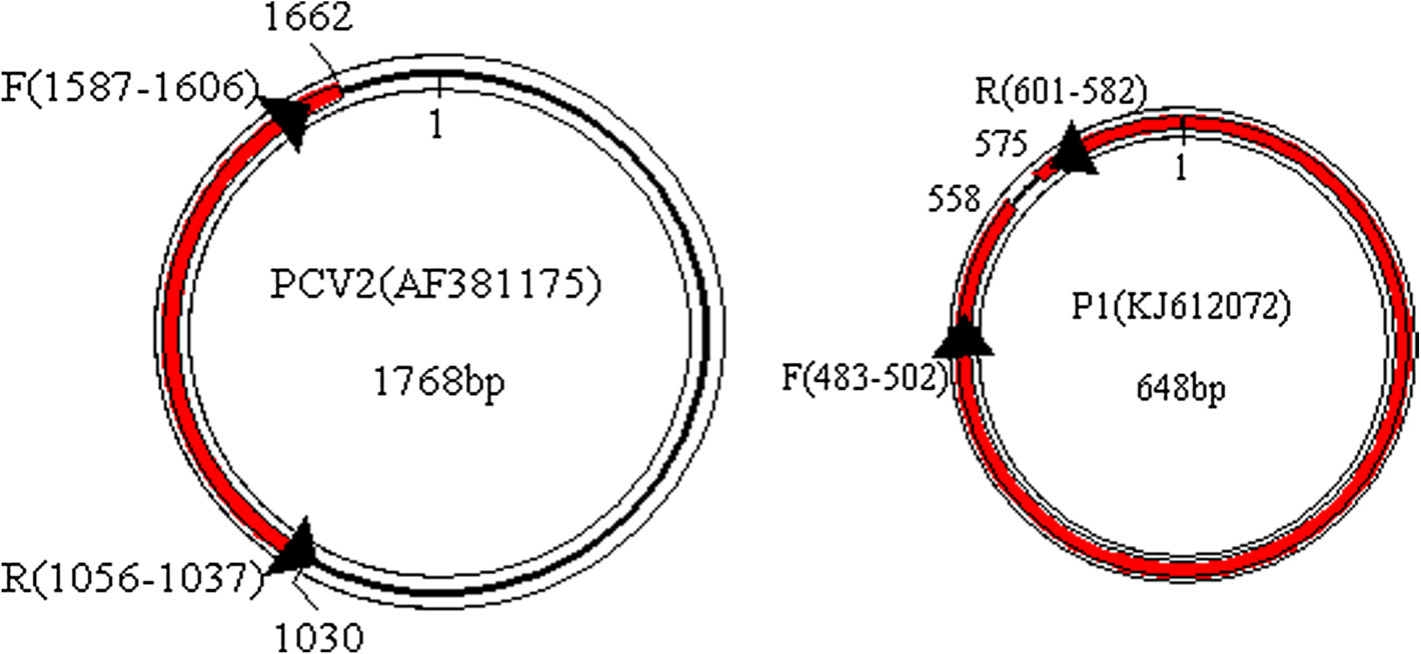
Genomic characterization of PCV2 and P1 and position of the primers in both genomes. The high homologous sequences of PCV2 and P1 are shown in bold, red colour, the primers in both genomes are indicated by the arrowheads, and their nucleotide coordinates are marked in both genomes

A pair of inverse primers was designed according to the P1 sequence [GeneBank:EF514716] using the software Oligo6.0. The sequence of the forward primer is 5′- GAGAGGCGGGTGTTGAAGAT-3 ′ and the reverse primer are 5′- AAGACCCCCCACTTAAACCC -3 ′. The amplification product is l19 bp for P1 and 1238 bp or 1237 bp for PCV2 based on different genome size (Fig. 1).

### Preparation of viral DNA template

PRV, PPV, PCV2 infected cell culture samples and clinical samples (including serum and stool) were used to prepare viral DNA samples. Among them, the stool samples were diluted 1:5 with sterile phosphate buffer (PBS), subjected to three freeze-thaw cycles, centrifuged and then the supernatant was used for viral DNA preparation. 300 μL of sample was mixed with 300 μL of cell lysis buffer [20 mmol/L Tris • Cl (pH8.0), 20 mmol/L EDTA (pH8.0), 0.5 % SDS (w/v)] and 10 μL of proteinase K (10 mg/mL), and then the sample was transferred to 50 °C to digest for 2 h. After digestion the sample was mixed with an equal volume of Tris-saturated phenol and centrifuged at 12000 r/min for 10 min. The upper aqueous phase was transferred to a new 1.5 mL eppendorf tube and mixed with an equal volume of phenol:chloroform:isoamyl alcohol (25:24:1) and centrifuged at 12000r/ min for 10 min. The upper aqueous phase was extracted with phenol: chloroform: isoamyl alcohol (25:24:1) one more time and was transferred to a new 1.5 mL eppendorf tube. Then the sample was mixed with 2 volumes of ice-cold ethanol and 1/10 volume of 3 mol/L sodium acetate (pH5.2) and sit at room temperature (20 − 25 °C) for 20 ~ 30 min to precipitate the genomic DNA. Following centrifugation at 12000 r/min for 10 min, the supernatant was discarded and the precipitates were washed in 70 % ethanol and air dried. DNA was dissolved in TE buffer containing 20 μg/mL RNaseA and stored at -20 °C for later use.

### Preparation of viral RNA template

Total RNA was extracted from PRRSV infected cells using TRIZOL LS Reagent according to the manufacturer’s instructions (Invitrogen). Reverse transcription was carried out immediately using the RNA sample. The reaction mix consisted of 11.5 μL RNA, 4 μL 5X buffer, 2 μL 10 mmol/L dNTPs Mixture, 0.5 μL 30 U/μL RNasin, 1 μL Oligo (dT)_18_, and 1 μL 10 U/μL AMV reverse transcriptase. After incubation at 42 °C for 1- 2 h, the resulting cDNA was stored at -20 °C for later use.

### Development of fluorescence quantitative standard curve

P1 genomic DNA was extracted from serum specimens of infected pigs and used as template for a 50 μL PCR reaction. The PCR conditions consisted of an initial denaturation at 95 °C for 5 min, followed by amplification for 35 cycles of 30 s at 95 °C, 30s at 60 °C, and 30 s at 72°Cfollowed by one cycle of 72 °C for 10 min. PCR product was separated on 2 % agarose gel by electrophoresis and recovered by agarose gel extraction. The DNA fragment was cloned into pMD18-T vector and transformed into *E. coli* TOP10. The resulting recombinant plasmid was verified by PCR, restriction enzyme digestion and DNA sequencing.

Plasmids with an OD 260 nm/OD 280 nm ratio between 1.8-2.0 were used to establish a standard curve. The concentration of the plasmids was calculated based on OD260nm value and used to calculate copy numbers. Six 10-fold serial dilutions of plasmid samples with copy number from 10^3^ to 10^8^ copies/μL were used as template to run reaction on the ABI 7500 quantitative PCR machine to generate a standard curve. The reaction mixture is 25 μL and contains 12.5 μL UltraSYBR Mix (2x) (CWBIOTEC products, China), 0.1 μM forward and reverse primers each, and 2 μL template. The reaction cycles are as follows: 95 °C for 10 min followed by 40 cycles of 95 °C for 15 s, 60 °C for 30s. After the final extension reaction added was the melting curve analysis. After completion of the reaction, a standard curve is automatically generated by the ABI software.

### Analysis of specificity, sensitivity and reproducibility of the SYBR I qPCR

To test the specificity of the method, PRV, PPV, PCV2, and P1 virus genomic DNA and PRRSV cDNA are used as templates in SYBR I qPCR according to the aforementioned reaction conditions. To test the sensitivity of the method, ten 10-fold serial dilutions of recombinant P1 DNA with concentration from 10^9^ to 10^0^ copies/μL are used as templates to run Real-time PCR. To test the reproducibility of the method, SYBR I qPCR reactions are repeated three times using 1:10 serial dilutions of P1 plasmids with copy numbers of 10^7^ to 10^4^ copies/μL and the obtained Ct values are compared.

### Clinical samples

368 sera samples and 6 manure samples were tested in this study. All samples were obtained from pig farms located in Jiangsu province, Anhui province, Beijing city and Tianjin city, respectively. Serum samples were collected in 2011 or 2012 from ~1 month old pigs with or without PMWS. Manure samples were collected in 2012 from piglets with severe diarrhea. The extracted DNAs from the aforementioned clinical samples were used as templates for SYBR I qPCR and also used as template for whole genome amplification of PCV2 and P1 by conventional PCR (Fenaux *et al.* [20]; Wen *et al.* [2]).

## Results

### PCR amplification of P1 gene fragment and construction of recombinant plasmid

P1 DNA was used as the template for PCR amplification and an 119 bp DNA fragment was obtained which is consistent with the size of the expected gene fragment. The obtained fragment was used to construct a recombinant plasmid which was verified by both PCR and restriction enzyme digestion analysis. Then the plasmid was verified by DNA sequencing.

### Generation of the standard curve and establishment of the linear regression equation

**Fig. 2:**
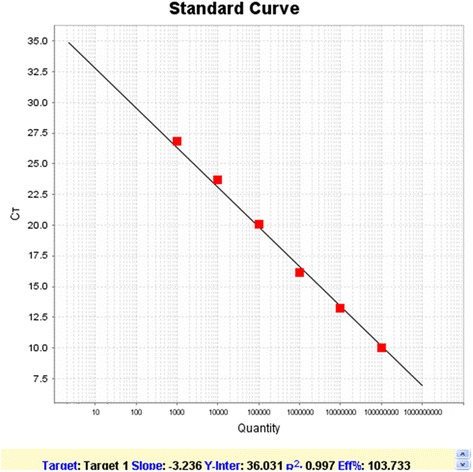
Standard curve of SYBR Green I fluorescence quantitative PCR detection of porcine circovirus-like agent P1 plasmid standards. X -axis is 10-fold serial dilutions of the plasmid standards (copies/μL), Y axis represents the threshold cycle (Ct) values

Six serial dilutions of plasmid with copy number from 10^3^ to 10^8^ copies/μL were used as template to run SYBR I qPCR. Following the reaction the system software automatically generated the amplification curves, melting curves, the standard curve and linear regression equation. The amplification graph exhibited an S-shape fluorescence amplification curve. The melting curve displayed a single peak and was consistent Tm values of approximately 83.0 °C. Standard curve analysis demonstrated that when copy numbers are in the range of 10^3^ to 10^8^ copies/μL, the copy numbers of P1 correlate linearly with Ct values with a slope of -3.236, the efficiency of the qPCR reaction as 103.7 %, and the value of the correlation coefficient R^2^ of the linear regression equation as 0.997 (Fig. 2) .

### Analysis of specificity, sensitivity and reproducibility of reaction

**Fig. 3:**
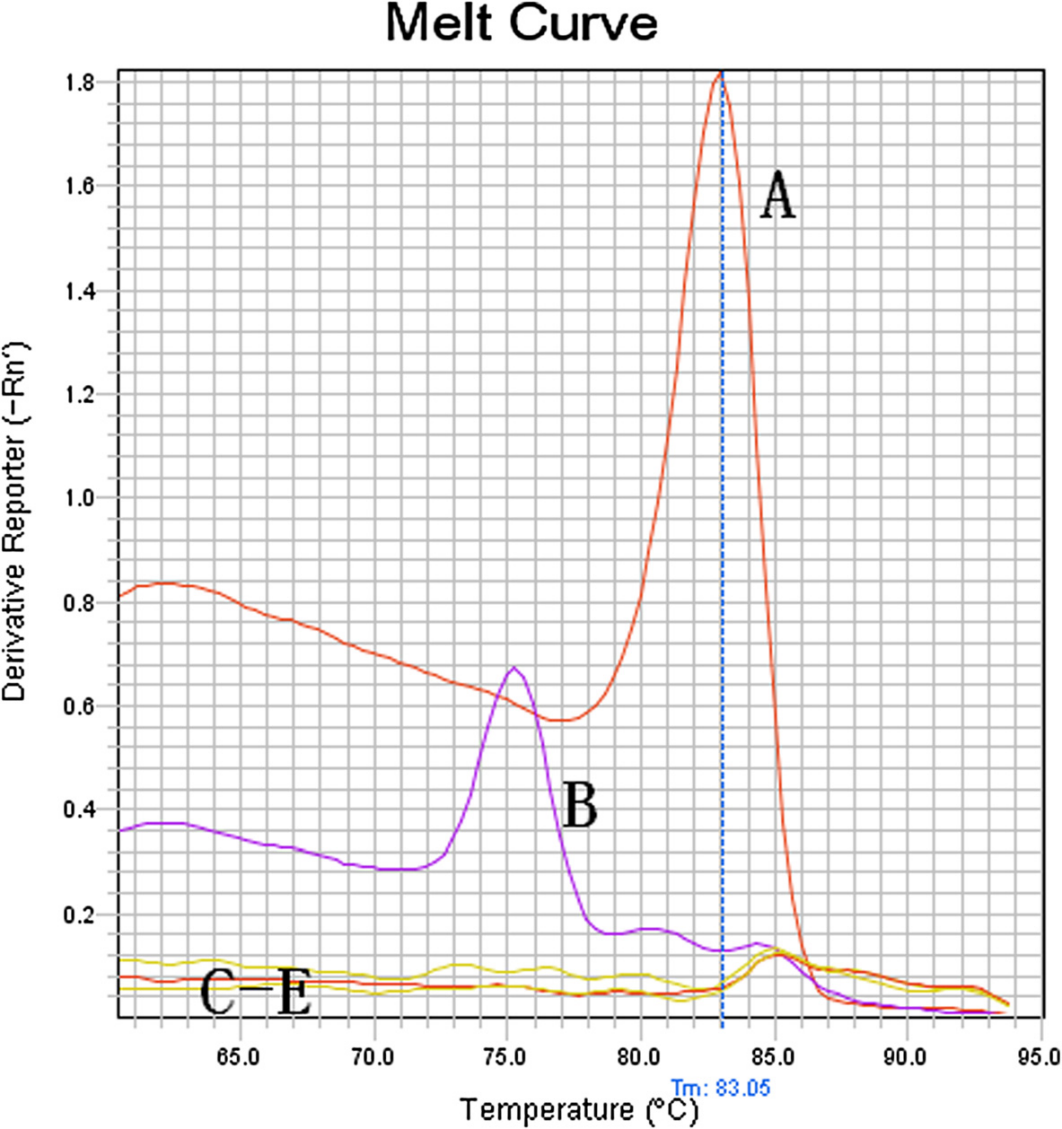
Specificity test of SYBR Green I fluorescence quantitative PCR detection of porcine circovirus-like agent P1. (*A*) P1; (*B*) PCV2; (*C*) PRV; (*D*) PRRSV; and (*E*) PPV

**Fig. 4:**
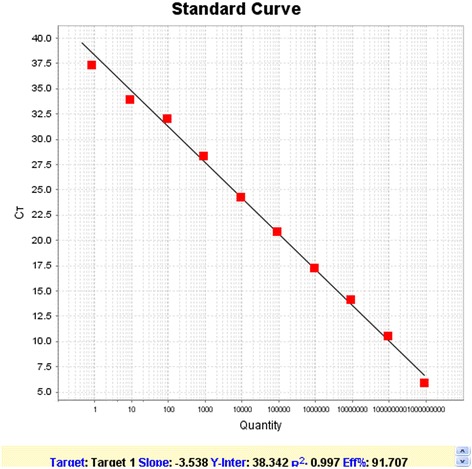
Sensitivity test of SYBR Green I fluorescence quantitative PCR detection of porcine circovirus-like agent P1. Plasmid standard was used as template, X -axis is 10-fold serial dilutions of the plasmid standards (copies/μl), Y axis represents the threshold cycle (Ct) values

SYBR I qPCR reactions using the P1 plasmid as a standard gave rise to the corresponding product and produced a single melting peak, indicating no primer dimers or other non-specific products amplified. The final product was further confirmed by DNA sequencing. Reaction using PCV2 or P1 as template generated a melting curve with a sharp peak, while the melting curve of PRV, PPV or PRRSV was flat. Although PCV2 gene also could be amplified, the melting temperature is approximately 75 °C which is different from that of P1. Thus the method has enough specificity (Fig. 3). Ten 10-fold serial dilutions of P1 with concentrations from 10^9^ to 10^0^ copies/μL were used as templates to run SYBR I qPCR The results showed that even at the low concentration of 10^0^ copies/μL, fluorescent signal could still be detected, indicating a high sensitivity and the concentration of templates correlates well with Ct values (Fig. 4). A Ct value of 40 was chosen as a cut-off value for standard positivity. Tests were repeated using the serial dilutions and further statistical analysis showed that the coefficient of variation within groups and between groups was less than 5 %, indicating good stability and reproducibility (Table 1).

**Table 1:** Reproducibility of a fluorescence-based real-time polymerase chain reaction for porcine circovirus-like virus P1

Copy number	Threshold cycle (Replicates of same batch of samples)			Coefficient of variation (%)	Threshold cycle (Replicates of different batches)			Coefficient of variation (%)
10^7^	12.48	12.51	12.50	0.12	12.50	13.26	12.74	3.04
10^6^	15.57	15.67	15.46	0.67	15.57	16.11	15.55	2.02
10^5^	19.10	18.98	18.94	0.44	19.01	20.10	19.11	3.12
10^4^	22.73	22.90	22.83	0.37	22.82	23.73	22.53	2.72

### Results of clinical sample analysis

By fluorescence-based quantitative PCR analysis 96 out of 368 serum samples and all six stool samples were identified as P1 positive (26 % of serum samples and 100 % of stool samples). P1 virus titers of the majority of samples (~70 %) were between 10^4^-10^5^ copies/mL with one manure sample up to 10^10^ copies/mL. The results are shown in Table 2. Out of 96 serum samples, 52 and 44 serum samples from healthy and PMWS pigs were postive for P1, respectively. The amount of P1 in healthy pigs were generally below P1 concentrations of 10^4^ genome copies/mL of total DNA, while the P1 concentrations in PMWS pigs were consistently higher than 10^5^ P1 DNA copies/mL of total DNA.

**Table 2:** P1 gene copy numbers of serum and fecal samples measured by Quantitative PCR

Sample type	Number of samples	Magnitude of P1 gene copy number (copies / mL)					
		10^3^	10^4^	10^5^	10^6^	10^7^	10^10^
Serum	368	7	37	30	15	7	0
Stool	6	0	2	1	2	0	1

The results of conventional PCR and quantitative PCR analysis of serum and manure samples were compared in Table 3. When applied to detection of PCV2 in the serum, conventional PCR identified 126 samples as PCV2 positive compared to the 108 samples by quantitative PCR. Neither conventional PCR nor quantitative PCR detected PCV2 in fecal samples. Conventional PCR can detect 52 serum samples as P1 positive compared to the 96 samples by quantitative PCR. Quantitative PCR detected 6 fecal samples as P1 positive compared to the 4 samples by conventional PCR. The quantitative PCR failed to detect the sixteen double positives found by conventional PCR. Collectively the results demonstrate that when the P1 gene copy number is less than 10^4^ copies/mL, it is difficult to use conventional PCR to detect.

**Table 3:** Comparison of results obtained by quantitative PCR (qPCR) and conventional PCR (PCR)

Sample type	Number of samples	PCV2 positive samples		P1 positive samples		PCV2 and P1 double positive samples	
Serum	368	qPCR	PCR	qPCR	PCR	qPCR	PCR
		108	126	96	85	0	16
Stool	6	0	0	6	4	0	0

## Discussion

Circoviridae is a family of single-stranded DNA viruses with a small genome size that can infect both poultry and pig. Two plant virus families, *Nanoviridae* and *Geminiviridae*, are considered to be closely related to Circoviridae. They all contain a stem-loop structure which encompasses a replication origin (Ori). Gibbs and Weiller, based on bioinformatics results of the Rep protein amino acid sequence of circovirus and Nanoviridae, provided a hypothesis of the origin and evolution of circovirus. In their study, they found that the N-terminal amino acid sequence of Rep in circovirus is homologous to that of *Nanoviridae* while the C-terminal amino acid sequence of the PCV Rep is closely related to RNA binding proteins 2C of vertebrate calicivirus. Therefore, they speculated that PCV is a combination of *Nanoviridae* and calicivirus. Since calicivirus is a RNA virus, the recombinant must involve a retrovirus or a retrotransposon [25].

Although the International Committee on Taxonomy of Viruses (ICTV) has not further classified viruses belonging to the same species, PCV2 could be divided into two major genotypes, namely PCV2a and PCV2b. Hesse *et al.* reported a PCV2 strain which contains a recombination of PCV2a ORF1 and PCV2b ORF2 [26]. Thereafter, PCV2a/b recombinants have been frequently isolated [27–29]. We identified porcine circovirus agent P1 during a study of PCV2 and suggested it may be a result of recombination of PCV2 with other molecules, likely with PCV2, because it also has circular single-stranded genome of 648 nucleotides. The 648 bp genome of P1 is the smallest DNA viral genome identified to date. Its genome is comprised of a DNA sequence highly homologous to PCV2 ORF2 as well as exogenous sequences^2^. The exogenous sequence of P1 is 16 nucleotides in size, and such a short fragment can be found in many species when a BLAST search was run to identify homologous sequence in GenBank, thus making it difficult to trace its origin. We can only speculate that P1 may be a product of a recombination between retrovirus and PCV2.

P1 infection of pigs can generate symptoms resembling PMWS, but these symptoms are different from symptoms of PCV2-associated PMWS in terms of anatomical and pathologic traits as well as viral distribution in tissues. The investigation of virus replication and pathological mechanism requires quantitative determination of virus copy numbers. The P1 genome has 648 nucleotides, and except ~10 nucleotides, its sequence is highly homologous to PCV2 ORF2 which contains 702 nucleotides. This indicates that the P1 genome is almost completely overlapped with PCV2 ORF2 and it is difficult for conventional PCR to distinguish between P1 and PCV2. In this study we designed a pair of primers which gave rise to a P1 PCR product of 119 bp and a PCV2 PCR product of 1238 (or 1237) bp. Thus, PCV2 will inevitably be amplified when using optimized quantitative PCR to detect P1, however its amplification efficiency and melting temperature are distinctively different from those of P1, providing a base to distinguish between the two viruses. The specificity of the SYBR I qPCR method was further confirmed by the fact that the study identified less positive samples than conventional PCR when applied to PCV2 test, as well as that it preferred to amplify P1 genome when DNA template contains both P1 and PCV2.

There are two commonly used quantitative real-time PCR techniques used, namely SYBR Green dye method and TaqMan probe method. SYBR Green I has advantages over the TaqMan technique; it avoids the use of fluorescent-labeled probes and other expensive reagents, and it is applicable to any PCR amplification system and can recognize primer dimers and nonspecific amplifications by melting curve analysis.

In this study a method to detect P1 based on SYBR I qPCR was successfully developed and the results show this approach is able to detect virus samples of a wide range of concentrations. When virus concentration falls between 10^0^ and 10^9^ copies/μL, PCR results correlate linearly with virus concentrations. With a high sensitivity the minimum virus concentration this method is able to detect is <10 copies/μL. This test method has no cross-reactivity with PPV, PRRSV, and PRV viruses and can distinguish PCV2 by melting curve analysis despite the fact that a small amount of PCV2 amplifications can also be detected, thus manifesting a high specificity. All results obtained by this method were very reproducible and the results of porcine serum samples showed that it can be easily applied to clinical analysis.

Even some healthy pigs were found to be P1 positive. And their serum P1 copy numbers were with the majority of them falling in between 10^4^ and 10^5^ copies/mL. Since P1 infection can cause PMWS-like symptoms to some pigs, the relationship between P1 copy numbers and symptoms needs further investigation. In 2010-2012 a serious infectious diarrhea disease affected a large number of pig farms in China and caused high rates of morbidity and mortality. No antibiotics or vaccines are effective to this disease and the economic loss was very significant. A new strain of porcine epidemic diarrhea virus was found to be the major pathogen, but porcine bocavirus and crest virus were also detected in clinical specimens. In this study high concentration of P1 virus was identified in all six stool samples obtained from diarrhea pigs, suggesting that P1 may play a role in this epidemic of diarrhea.

In previous studies using Real-time quantitative PCR assay to detect PCV2, either dye method [30] or probe method [31, 32], ORF2 gene was selected as the target. This method cannot accurately measure PCV2 concentration under circumstances of P1 and PCV2 double infection. P1 can only be distinguished when PCV2 ORF1 is selected as the assay target [33]. Recent studies have shown that PCV2 can form intramolecular rearrangements of variable size which also have circular single-stranded genome consisting of nucleotide sequences derived entirely from the parent strains. These molecules do not contain exogenous nucleotides and their cleavage sites are different from those of PCV2 [34, 35]. Some rearrangements contain a part of ORF1 fragment and rearrangements which contains longer fragment of ORF1 also likely occur. The emergence of PCV2 molecular rearrangements challenges the validity of quantitative PCR diagnostic methods based on ORF1.

## Conclusions

In summary, this study established a method with high specificity, sensitivity and reproducibility to detect P1 based on SYBR Green I real-time quantitative PCR. This method can determine P1 copy number even in the presence of PCV2 and P1 double positive samples, rendering it possible to monitor *in vivo* P1 replication status. Thus our study paved a way for studies of P1 infection and proliferation, and for the study of the pathogenesis and prevention of P1. The emerging PCV2 molecular rearrangements have brought trouble to P1 and PCV2 quantification. In future studies, amplification of full-length genome by conventional PCR followed by DNA sequencing to exclude rearrangements should be applied to accurately quantify P1 and PCV2.

## Abbreviations

PCV2: Porcine circovirus type 2
P1: Porcine circovirus-like virus P1
PRV: Pseudo-rabies virus
PPV: Porcine parvovirus
PRRSV: Porcine reproductive and respiratory syndrome virus
PMWS: Postweaning multisystemic wasting syndrome
qPCR: Real-time Quantitative PCR Detecting System
Ct: Threshold cycle
